# Ghrelin

**DOI:** 10.1016/j.molmet.2015.03.005

**Published:** 2015-03-21

**Authors:** T.D. Müller, R. Nogueiras, M.L. Andermann, Z.B. Andrews, S.D. Anker, J. Argente, R.L. Batterham, S.C. Benoit, C.Y. Bowers, F. Broglio, F.F. Casanueva, D. D'Alessio, I. Depoortere, A. Geliebter, E. Ghigo, P.A. Cole, M. Cowley, D.E. Cummings, A. Dagher, S. Diano, S.L. Dickson, C. Diéguez, R. Granata, H.J. Grill, K. Grove, K.M. Habegger, K. Heppner, M.L. Heiman, L. Holsen, B. Holst, A. Inui, J.O. Jansson, H. Kirchner, M. Korbonits, B. Laferrère, C.W. LeRoux, M. Lopez, S. Morin, M. Nakazato, R. Nass, D. Perez-Tilve, P.T. Pfluger, T.W. Schwartz, R.J. Seeley, M. Sleeman, Y. Sun, L. Sussel, J. Tong, M.O. Thorner, A.J. van der Lely, L.H.T. van der Ploeg, J.M. Zigman, M. Kojima, K. Kangawa, R.G. Smith, T. Horvath, M.H. Tschöp

**Affiliations:** 1 Institute for Diabetes and Obesity, Helmholtz Zentrum München, München, Germany; 2 Department of Physiology, Centro de Investigación en Medicina Molecular y Enfermedades Crónicas, University of Santiago de Compostela (CIMUS)-Instituto de Investigación Sanitaria (IDIS)-CIBER Fisiopatología de la Obesidad y Nutrición (CIBERobn), Santiago de Compostela, Spain; 3 Division of Endocrinology, Department of Medicine, Beth Israel Deaconess Medical Center, Boston, MA, USA; 4 Department of Physiology, Faculty of Medicine, Monash University, Melbourne, Victoria, Australia; 5 Applied Cachexia Research, Department of Cardiology, Charité Universitätsmedizin Berlin, Germany; 6 Department of Pediatrics and Pediatric Endocrinology, Hospital Infantil Universitario Niño Jesús, Instituto de Investigación La Princesa, Madrid, Spain; 7 Department of Pediatrics, Universidad Autónoma de Madrid and CIBER Fisiopatología de la obesidad y nutrición, Instituto de Salud Carlos III, Madrid, Spain; 8 Centre for Obesity Research, University College London, London, United Kingdom; 9 Metabolic Disease Institute, Division of Endocrinology, Department of Medicine, University of Cincinnati College of Medicine, Cincinnati, OH, USA; 10 Tulane University Health Sciences Center, Endocrinology and Metabolism Section, Peptide Research Section, New Orleans, LA, USA; 11 Division of Endocrinology, Diabetes and Metabolism, Dept. of Medical Sciences, University of Torino, Torino, Italy; 12 Department of Medicine, Santiago de Compostela University, Complejo Hospitalario Universitario de Santiago (CHUS), CIBER de Fisiopatologia Obesidad y Nutricion (CB06/03), Instituto Salud Carlos III, Santiago de Compostela, Spain; 13 Duke Molecular Physiology Institute, Duke University, Durham, NC, USA; 14 Translational Research Center for Gastrointestinal Disorders, University of Leuven, Leuven, Belgium; 15 New York Obesity Nutrition Research Center, Department of Medicine, St Luke's-Roosevelt Hospital Center, Columbia University College of Physicians and Surgeons, New York, NY, USA; 16 Department of Pharmacology & Molecular Sciences, The Johns Hopkins University School of Medicine, Baltimore, MD, USA; 17 Monash Obesity & Diabetes Institute, Monash University, Clayton, Victoria, Australia; 18 Division of Metabolism, Endocrinology and Nutrition, Department of Medicine, University of Washington School of Medicine, Seattle, WA, USA; 19 McConnell Brain Imaging Centre, Montreal Neurological Institute, McGill University, Montreal, Quebec, Canada; 20 Dept of Neurobiology, Yale University School of Medicine, New Haven, CT, USA; 21 Department of Physiology/Endocrinology, Institute of Neuroscience and Physiology, The Sahlgrenska Academy at the University of Gothenburg, Gothenburg, Sweden; 22 Department of Physiology, School of Medicine, Instituto de Investigacion Sanitaria (IDIS), University of Santiago de Compostela, Spain; 23 Department of Psychology, Institute of Diabetes, Obesity and Metabolism, University of Pennsylvania, Philadelphia, PA, USA; 24 Department of Diabetes, Obesity and Metabolism, Oregon National Primate Research Center, Oregon Health & Science University, Beaverton, OR, USA; 25 Comprehensive Diabetes Center, University of Alabama School of Medicine, Birmingham, AL, USA; 26 Division of Diabetes, Obesity, and Metabolism, Oregon National Primate Research Center, Oregon Health and Science University, Beaverton, OR 97006, USA; 27 NuMe Health, 1441 Canal Street, New Orleans, LA 70112, USA; 28 Departments of Psychiatry and Medicine, Brigham and Women's Hospital and Harvard Medical School, Boston, MA, USA; 29 Department of Neuroscience and Pharmacology, University of Copenhagen, Copenhagen N, Denmark; 30 Department of Psychosomatic Internal Medicine, Kagoshima University Graduate School of Medical and Dental Sciences, Kagoshima, Japan; 31 Institute of Neuroscience and Physiology, The Sahlgrenska Academy at the University of Gothenburg, Gothenburg, Sweden; 32 Medizinische Klinik I, Universitätsklinikum Schleswig-Holstein Campus Lübeck, Lübeck, Germany; 33 Centre for Endocrinology, William Harvey Research Institute, Barts and the London, Queen Mary University of London, London, UK; 34 New York Obesity Research Center, Department of Medicine, Columbia University College of Physicians and Surgeons, New York, NY, USA; 35 Diabetes Complications Research Centre, Conway Institute, University College Dublin, Ireland; 36 Division of Neurology, Respirology, Endocrinology and Metabolism, Department of Internal Medicine, Faculty of Medicine, University of Miyazaki, Kiyotake, Miyazaki, Japan; 37 Division of Endocrinology and Metabolism, University of Virginia, Charlottesville, VA, USA; 38 Department of Internal Medicine, Department of Medicine, University of Cincinnati College of Medicine, Cincinnati, OH, USA; 39 Department of Neuroscience and Pharmacology, Laboratory for Molecular Pharmacology, The Panum Institute, University of Copenhagen, Copenhagen, Denmark; 40 Department of Surgery, University of Michigan School of Medicine, Ann Arbor, MI, USA; 41 Children's Nutrition Research Center, Department of Pediatrics, Baylor College of Medicine, Houston, TX, USA; 42 Department of Genetics and Development, Columbia University, New York, NY, USA; 43 Department of Medicine, Erasmus University MC, Rotterdam, The Netherlands; 44 Rhythm Pharmaceuticals, Boston, MA, USA; 45 Departments of Internal Medicine and Psychiatry, The University of Texas Southwestern Medical Center, Dallas, TX, USA; 46 Molecular Genetics, Institute of Life Science, Kurume University, Kurume, Japan; 47 National Cerebral and Cardiovascular Center Research Institute, Osaka, Japan; 48 The Scripps Research Institute, Florida Department of Metabolism & Aging, Jupiter, FL, USA; 49 Program in Integrative Cell Signaling and Neurobiology of Metabolism, Section of Comparative Medicine, Yale University School of Medicine, New Haven, CT, USA; 50 Division of Metabolic Diseases, Department of Medicine, Technical University Munich, Munich, Germany

**Keywords:** Ghrelin, Growth hormone segretagogue receptor

## Abstract

**Background:**

The gastrointestinal peptide hormone ghrelin was discovered in 1999 as the endogenous ligand of the growth hormone secretagogue receptor. Increasing evidence supports more complicated and nuanced roles for the hormone, which go beyond the regulation of systemic energy metabolism.

**Scope of review:**

In this review, we discuss the diverse biological functions of ghrelin, the regulation of its secretion, and address questions that still remain 15 years after its discovery.

**Major conclusions:**

In recent years, ghrelin has been found to have a plethora of central and peripheral actions in distinct areas including learning and memory, gut motility and gastric acid secretion, sleep/wake rhythm, reward seeking behavior, taste sensation and glucose metabolism.

## 1 Introduction

**Figure 1:**
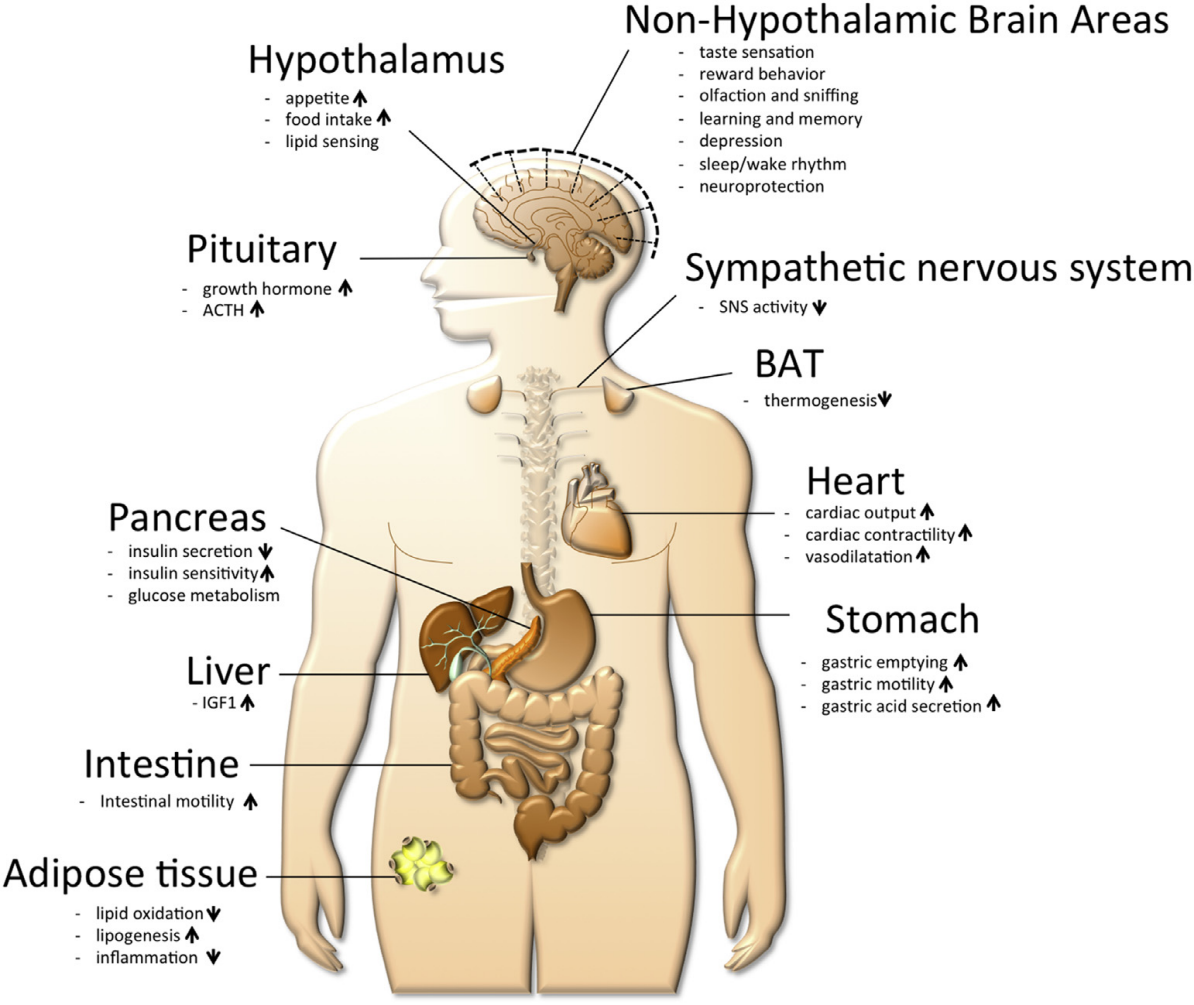
Schematic on ghrelin's physiological effects.

In 1999, Masayasu Kojima, Kenji Kangawa, and colleagues discovered the gastrointestinal peptide hormone ghrelin as the endogenous ligand for the growth hormone secretagogue receptor (GHSR)1a, capable of stimulating growth hormone (GH) release from the anterior pituitary gland [1]. In 2000, Mark Heiman and Matthias Tschöp discovered that ghrelin acts in the brain to regulate food intake, body weight, adiposity, and glucose metabolism [2]. Ghrelin was found to modulate systemic metabolism via activation of orexigenic neural circuits [3,4]. Subsequently, numerous central and peripheral actions of ghrelin were described, including stimulation of gut motility and gastric acid secretion [5,6], modulation of sleep [7–9], taste sensation and reward seeking behavior [10–16], regulation of glucose metabolism [17–20], suppression of brown fat thermogenesis [21–25], modulation of stress and anxiety [26–28], protection against muscle atrophy [29,30], and improvement of cardiovascular functions such as vasodilatation and cardiac contractility [31–34] (Figure 1).

In the early stages of ghrelin research, a model emerged suggesting that ghrelin acts as a “meal initiation” or “hunger“ hormone, signaling gastrointestinal (GI) fuel status to the central nervous system (CNS) in order to adjust food intake and energy expenditure [3,35–38]. Consistent with this role, ghrelin is produced in the oxyntic glands of the gastric fundus [1], its blood levels rise with increased hunger sensations [36,39], and its receptor is located in the hypothalamic neurons that regulate food intake and satiety [40–42]. Recently, however, this traditional and narrowly defined view of ghrelin as a “hunger hormone” has been challenged. Increasing evidence supports a more complex role for ghrelin in the regulation of hunger and metabolism. The aim of this review is to examine the variety of biological functions of ghrelin in order to emphasize its multifaceted nature and to answer some questions that persist after 15 years of ghrelin research.

## 2 Discovery of ghrelin as the endogenous ligand of the growth hormone secretagogue receptor 1a (GHSR1a)

In the late 1970s, the work of Cyril Bowers and Frank Momany led to the generation of a group of synthetic opioid peptide derivatives that promoted the release of GH from the anterior pituitary [43,44]. The molecules, which Bowers and Momany referred to as GH releasing peptides (GHRPs), were generated by the chemical modification of met-enkephalin and included growth hormone releasing peptide (GHRP)-6, GHRP-2, and hexarelin [45]. Initially, it was thought that these GHRPs acted only on the pituitary, but soon it became clear that that they also acted on the hypothalamic arcuate nucleus (ARC) [46], specifically on GH-releasing hormone (GHRH) neurons [41]. The mechanism by which these molecules promoted the release of GH was unknown, but it was distinct from that of the GHRH/somatostatin pathway [46–49]. In 1996, the GHS clinical candidate MK0677 was employed by Roy Smith and Lex van der Ploeg to clone the GH secretagogue receptor (GHSR1a) [50], at which GHSs and GHRPs were shown to be agonists.

In humans, the *GHSR1* gene codes for the full-length G-protein coupled seven transmembrane protein GHSR1a, but a truncated isoform (GHSR1b), which has a wide tissue distribution, is also transcribed [51]. GHSR1a has been shown to homodimerize, but the possibility has been raised that GHSR1a and GHSR1b also heterodimerize [52,53] and that the heterodimer inhibits the activation of GHSR1a [53].

GHSR1a is expressed predominantly in the anterior pituitary gland, pancreatic islets, adrenal gland, thyroid, myocardium, ARC, hippocampus, the substantia nigra pars compacta (SNpc), ventral tegmental area (VTA), and raphe nuclei [40,51]. In the ARC, in addition to being expressed in GHRH neurons, *GHSR1a* is colocalized in neurons that express neuropeptide Y (*Npy*) and Agouti related peptide (*Agrp*), which regulate food intake and satiety [42]. Along with the observation that GHRP-6 induced activation of GHSR1 and increased c-Fos expression in NPY neurons [41], these data suggested the presence of an unknown but endogenous ligand for GHSR1, one that might regulate systemic metabolism.

In the years that followed, extensive research efforts were aimed at identifying the endogenous ligand for GHSR1. The ligand remained elusive until 1999 when Kojima and colleagues identified the cognate agonist for GHSR1. Purified from rat stomach extracts, the 28 amino acid peptide was named ‘ghrelin’, a name originating from ‘ghre’, the Proto-Indo-European root of the word 'grow' [1].

## 3 Regulation of ghrelin acylation

### 3.1 Acylation of ghrelin by the ghrelin O-acyl-transferase (GOAT)

**Figure 2:**
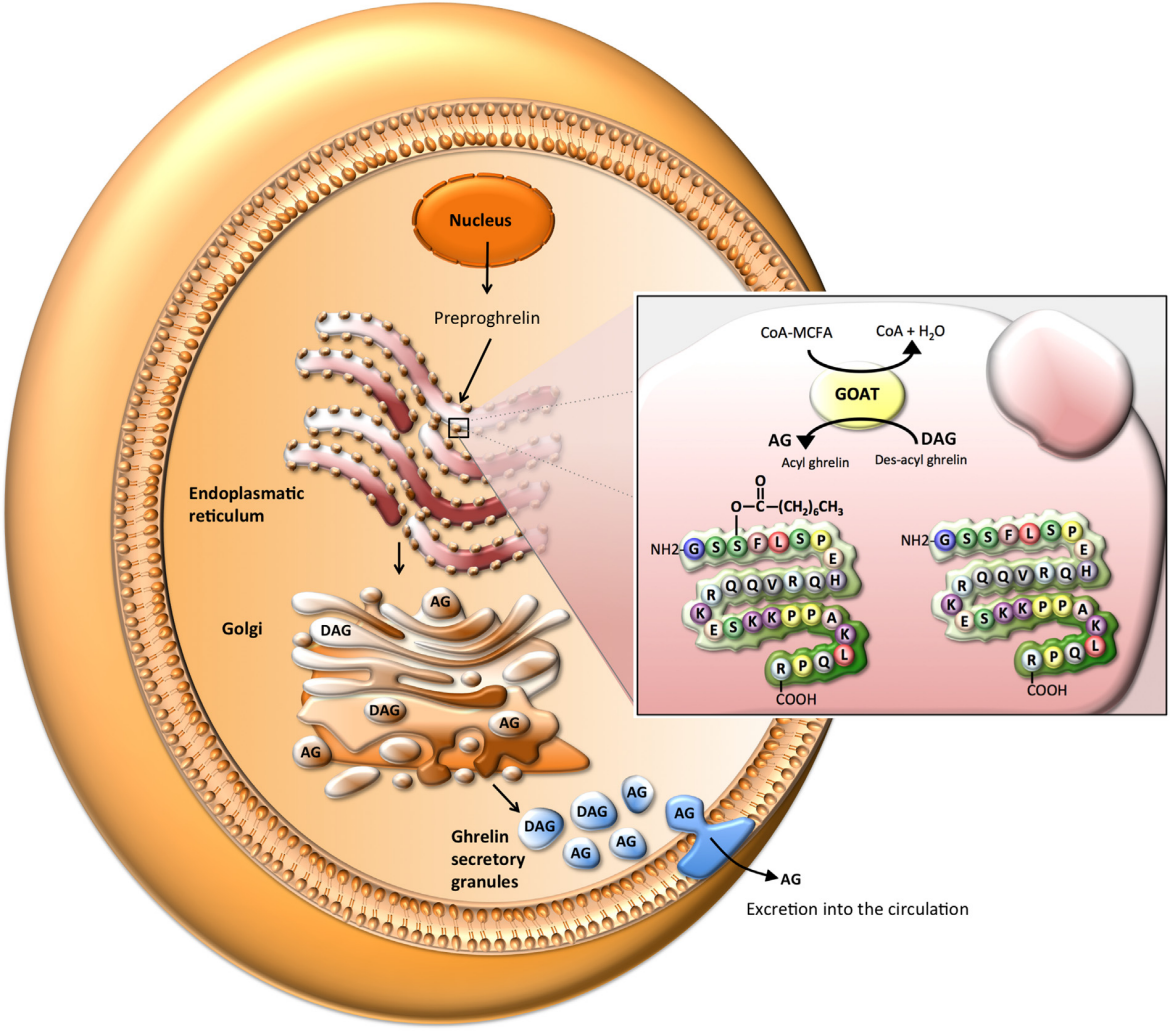
Schematic on the post-translational processing and acylation of ghrelin.

Ghrelin is encoded by the preproghrelin gene (Figure 2), which, in addition to ghrelin, also encodes for a small signal peptide and the 23 amino acid peptide obestatin. Originally, it was thought that obestatin was the endogenous ligand for GPR39 and could inhibit food intake and gastric motility, functions that could counteract the effect of ghrelin [54]. However, several independent groups could not confirm these findings and identified Zn^2+^ as a physiological agonist of GPR39 [55]. To activate its only known receptor, ghrelin requires the attachment of a fatty acid side-chain (preferably C8 or C10) to its serine 3 residue, a rare post-translational modification (acylation) that is achieved by the ghrelin *O*-acyl-transferase (GOAT), a member of the membrane-bound *O*-acyltransferase (MBOAT) family [56,57] (Figure 2).

**Figure 3:**
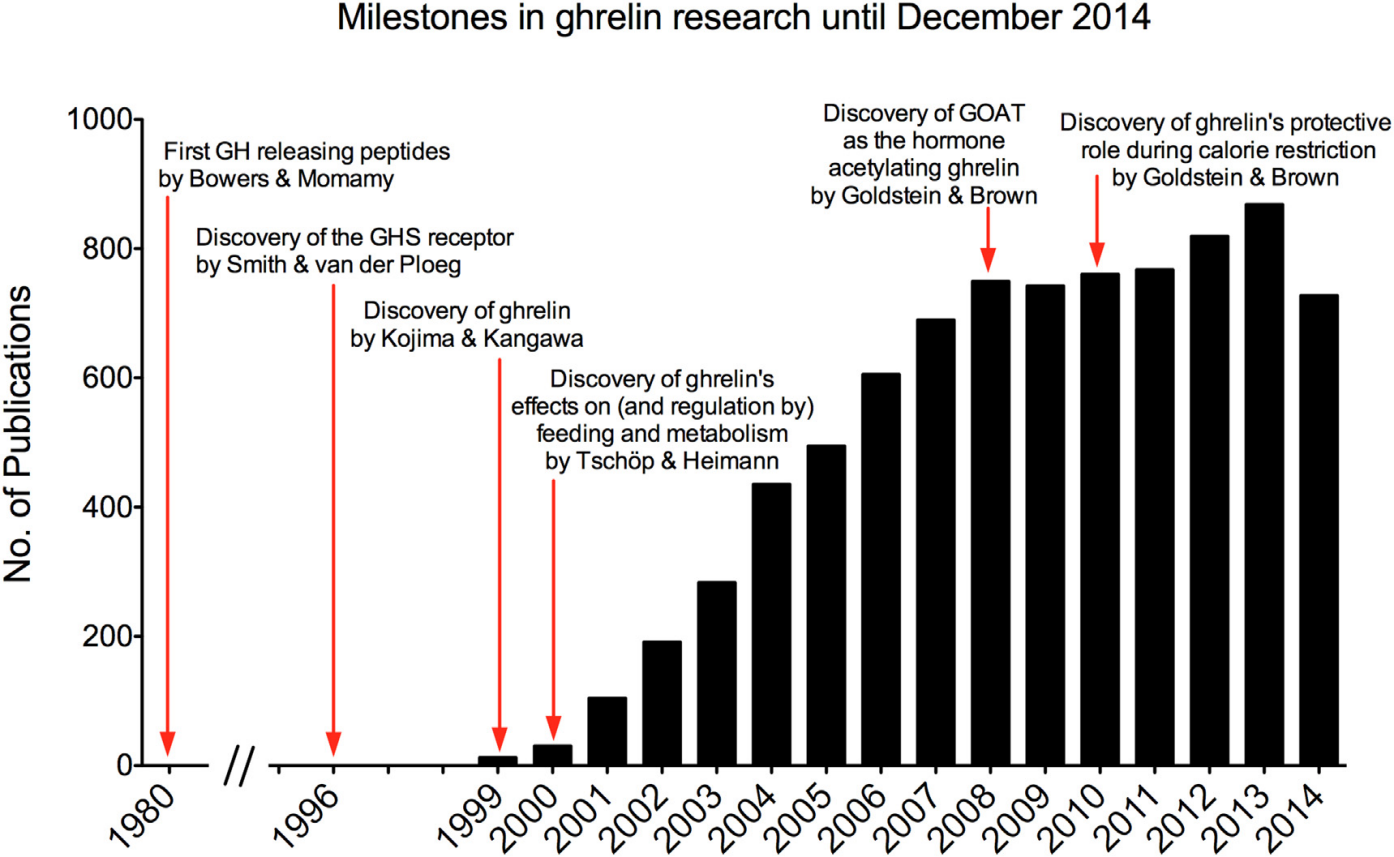
Milestones in ghrelin research. Bar graph represents the number of publications listed in the US National Library of Medicine National Institute of Health (PubMed) and that contain the word ‘ghrelin’ in either the title or the abstract until December 2014.

The discovery of GOAT as the enzyme responsible for ghrelin acylation [56,57] has been a major breakthrough for understanding the role that acyl-modification plays in ghrelin's physiology (Figure 3). This modification, mainly octanoylation and, to a lesser extent, decanoylation, is required for ghrelin's effects on systemic metabolism. The data demonstrating GOAT's essential role in the activation of ghrelin are clear. First, GOAT and des-acyl ghrelin are sufficient to recapitulate the production of acyl-modified ghrelin in cells that normally do not express either of these gene products [56,57]. Second, ghrelin and GOAT share a similar tissue expression profiles in both humans and mice with highest GOAT expression in pancreas and stomach in humans and the stomach and intestine in mice [56,58,59]. Third, GOAT, like ghrelin, is highly conserved across vertebrates. Humans, rats, mice, and zebrafish all exhibit functional GOAT activity, and sequences with amino acid similarities to GOAT are present in other vertebrates, consistent with the presence of octanoylated forms of ghrelin across vertebrates [56]. Finally, the most convincing data for GOAT as ghrelin's acyl transferase are from *GOAT*-deficient mice, which completely lack octanoyl and decanoyl modified forms of ghrelin [20,56,60–62].

### 3.2 Substrates for GOAT-mediated ghrelin acylation

Intriguingly, the lipids used for ghrelin activation are, at least in part, directly recruited from the pool of ingested dietary lipids [61,63] in a process that may take advantage of the fact that ghrelin-producing X/A-like cells are located within gastric oxyntic glands. A significant number of these cells are apposed to the stomach lumen, allowing for direct access to a supply of dietary lipids [64]. Furthermore, the preferred fatty acid substrates for GOAT are derived from medium-chain-triglycerides, which can be directly absorbed into the circulation without being broken down by lipases and bile acids [65]. Despite this evidence, the relative contribution of *de novo* synthesized fatty acids in comparison to those directly derived from the diet as substrate of GOAT for ghrelin acylation remains unknown. Mutation studies in the region of the acylated serine 3 have revealed that glycine 1, serine 3, and phenylalanine 4 are critical components of the recognition sequence for GOAT, whereas serine 2, leucine 5, serine 6 and proline 7 seem to be less important [62].

Biochemically, GOAT appears to have two critical substrates, des-acyl ghrelin and short-to mid-chain fatty acids thioesterified with Coenzyme A. Cells expressing both ghrelin and GOAT synthesize serine 3 acyl-ghrelin, with the acyl moiety precursors derived from fatty acids ranging from acetate (C2) to tetradecanoic acid (C14) [56]. The length of the fatty acid used for ghrelin acylation seems to be of importance for ghrelin's metabolic effects, as alterations in the fatty acid length result in differential activation of GHSR1a *in vitro* and alter ghrelin's effect on food intake and adiposity *in vivo* [66]. Thus, modulation of the acyl side-chain may also represent an interesting therapeutic control point for future interventions.

Octanoyl- and decanoyl-modified ghrelin forms are the optimal ligands for activation of the GHSR1a [57,62]. *In vitro* studies recreating the acyl-modification of ghrelin with des-acyl ghrelin peptides, fatty acid CoA esters, and GOAT containing microsomes define the substrate specificity for GOAT. These studies support the idea that GOAT requires fatty acid substrates as high energy fatty acid CoA thioesters and that the amino acid sequence GXSFX, where G, X, S, and F correspond to unblocked amino terminal glycine (G), any amino acid (X), serine (S) and phenylalanine (F), respectively, is sufficient as a substrate for GOAT acylation [67]. The structural constraints defined by this amino acid motif appear specific only for ghrelin and suggest that ghrelin may be the principal peptide substrate for GOAT. Most recent studies comparing the *in vitro* selectivity of hexanoyl- and octanoyl-CoA substrates suggest that GOAT may actually prefer hexanoyl CoA over octanoyl CoA substrates, highlighting the importance of the specific fatty acid metabolism in acyl ghrelin producing cells, responsible for producing circulating levels of octanoyl and decanoyl ghrelin [67].

### 3.3 Evidence suggesting a role of the GOAT-ghrelin system as a nutrient sensor

Most recent studies with genetically modified mice, which are either lacking GOAT or overexpressing both ghrelin and GOAT, establish that the GOAT-ghrelin system acts as a nutrient sensor informing the body of the presence of nutrients, rather than the absence, as commonly proposed [61]. Several observations support this statement. First, prolonged fasting of mice led to well-established, increased levels of total ghrelin, which were caused by increased des-acyl ghrelin rather than acyl ghrelin. This increase in des-acyl ghrelin occurred as GOAT transcript levels decreased in response to the prolonged fasting treatments [61]. Consistent with these observations, GOAT-null mice showed significantly increased total ghrelin levels, being driven only by des-acyl ghrelin as these mice are unable to produce acyl modified ghrelin [56,61]. Second, several studies showed that dietary medium-chain fatty acids (MCFAs) can be a direct source of substrates for ghrelin acylation in rodents and that sensing of MCFAs involves the gustatory G-protein, α-gustducin [61,63,68]. Third, studies also show that mice lacking *GOAT* have lower body weight and fat mass on a MCFA-containing diet compared to wt mice, whereas transgenic mice overexpressing ghrelin and *GOAT* show higher body weight and fat mass and decreased energy expenditure than wt littermates, demonstrating a role for the endogenous acyl-ghrelin in the control of energy balance and adiposity. In addition, data show that a sufficient dietary supply of medium chain triglycerides is crucial for ghrelin acylation, since ghrelin and *GOAT* overexpressing mice are unable to produce large amounts of octanoylated ghrelin when fed a low fat carbohydrate-rich chow diet. Interestingly, transgenic mice fed a regular chow diet show substantial amounts of inactive C2-acetyl–modified ghrelin in the absence of octanoylated ghrelin, suggesting that, at least under these experimental conditions, the GOAT fatty acid substrate for acylation, acetyl-CoA, is sufficiently available for ghrelin acylation. Dietary supplementation of octanoyl triglycerides increases octanoyl-modified ghrelin in these ghrelin and GOAT transgenic animals [61].

Based on these data, it is likely that the GOAT-ghrelin system acts as a nutrient sensor by using readily absorbable MCFAs to signal to the brain that high caloric food is available, leading to optimization of nutrient partitioning and growth signals [61,63]. These recent observations, while informative on the regulation of ghrelin's function by GOAT, highlight key questions that need to be resolved. First, are the observations on the role of GOAT and ghrelin in nutrient sensing and the endocrine control of energy expenditure translatable to humans? Second, what is the physiological role and what are key players for this proposed acyl-ghrelin feedback mechanism observed in the GOAT-deficient animals? Finally, what is (are) the specific biochemical pathway(s) in ghrelin and GOAT expressing cells that produce the necessary levels of C8-CoAs critical for the synthesis of physiologically relevant octanoylated ghrelin? Understanding these fundamental aspects for ghrelin and GOAT will provide critical new insights on the physiological function of this pathway on human physiology.

## 4 Biological functions of ghrelin

### 4.1 Clinical pharmacology studies on ghrelin's effect on energy metabolism

Numerous human studies have evaluated the effect of ghrelin and its analogs on GH secretion [69–72], food intake [11,34,73–79], body weight [34,78,80] energy expenditure [81–83], glucose homeostasis [84–89], and gastrointestinal motility [90–93]. Peripheral ghrelin or GHRP-2 administration reliably induces the sensation of hunger and increases food intake in lean, obese, healthy and malnourished individuals [94]. Interestingly, iv administration of ghrelin in healthy volunteers increases neural activity in specific brain regions in response to pictures of food. Endogenous fasting ghrelin is positively related to hunger-modulated activity in the hypothalamus, amygdala, and prefrontal cortex in response to palatable food stimuli [95,96]. Activation of these reward centers by ghrelin suggests enhancement of food consumption is a more complex mechanism than the physical sensations of hunger or satiety [97,98]. Interestingly, these ghrelin-brain activity relationships are absent in women with anorexia nervosa, suggesting the possibility of CNS-regulated ghrelin resistance in these individuals [95].

### 4.2 Clinical pharmacology studies on ghrelin's effect on the GH-Axis

In humans, a single intravenous (iv) injection or a continuous 24 h ghrelin infusion induces acute GH release [99,100] and increases 24 h pulsatile GH secretion [101]. The importance of ghrelin in GH regulation is supported by the observation that abnormalities of GHSR function may be associated with familial short stature [102]. The GHSR locus is one of the top sites suggested to contribute to the genetic variation of height [103]. Several studies have assessed the association of ghrelin and GHSR single nucleotide polymorphisms (SNPs) with height under conditions of obesity and diabetes [reviewed in [104]]. At high doses, ghrelin also increases levels of adrenocorticotropic hormone (ACTH), prolactin, and cortisol levels [70], while it inhibits levels of luteinizing hormone (LH) [105]. These effects desensitize and normalize with prolonged ghrelin (or ghrelin mimetic) treatment [45,106]. Furthermore, ghrelin mimetics have been investigated as diagnostic agents to establish growth hormone deficiency [107] as well as a therapeutic option for age-dependent GH decline and have yielded some potentially beneficial effects [80] (Table 1). Notably, whereas several clinical studies support a role of ghrelin in regulation growth and height, mice lacking GHSR, ghrelin or GOAT show no growth abnormalities. Whether these discrepant results from mice and humans speaks for distinct GH-release pathways as of today elusive.

### 4.3 Clinical pharmacology studies on ghrelin's effect on glucose metabolism

Both ghrelin and its receptor are widely expressed in multiple regions of the brain [3,108,109] and in peripheral tissues, such as the intestine [110], pituitary [111,112], kidney [108,113], lung [108,114], heart [110,115,116], ovaries [108], and pancreatic islets [17,40]. Expression in pancreatic islets is consistent with a series of human studies showing increased plasma levels of glucose and decreased plasma levels of insulin following ghrelin administration [84–86,88,89]. GHSRs are also expressed by α-cells of the pancreatic islet and likely contribute to the ability of ghrelin to directly stimulate glucagon secretion [117].

Ghrelin inhibits insulin secretion in most animal studies [118–121], and blockade of pancreatic-derived ghrelin enhances insulin secretion and ameliorates the development of diet-induced glucose intolerance [122]. Supporting these data is the finding that plasma ghrelin and insulin levels seem to be negatively correlated, as the two hormones exhibit reciprocal changes during the day and during a hyperinsulinemic, euglycemic clamp [37,123]. Furthermore, continuous ghrelin infusion for 65 min suppresses glucose-stimulated insulin secretion and impairs glucose tolerance in healthy individuals [124]. In line with this, pharmacological inhibition of GOAT improves glucose disposal by stimulating the release of insulin [60]. The reciprocal relationship of ghrelin and insulin is supported by epidemiologic studies showing an inverse relationship between circulating ghrelin levels and indexes of insulin resistance [39]. A single iv dose of ghrelin significantly increases plasma glucose levels followed by a reduction in fasting insulin levels in lean [84] and obese subjects with or without polycystic ovarian syndrome [87], suggesting inhibition of insulin secretion. Intriguingly, ghrelin's suppression of insulin secretion in pancreatic β-cells is mediated by a non-canonical GHSR1a signaling pathway in which Gα_i_ rather than Gα_q_ is coupled to the receptor [125]. This modified signaling is dependent upon agonist mediated molecular interactions between GHSR1a and somatostatin receptor subtype-5 (SST5) and the formation of GHSR1a:SST5 heterodimers [126]. Conversely, some studies suggest that ghrelin has positive trophic activity, protecting from β-cell damage in experimental models of type 1 diabetes [127,128]. However, the onset of type 1 diabetes is associated with decreased circulating ghrelin levels [129,130].

### 4.4 Clinical pharmacology studies on ghrelin's effect on GI-Motility

Very shortly after the discovery of ghrelin by Kojima and Kangawa, ghrelin was also described by another group, who named it motilin-related peptide because of its homology with motilin, a gut hormone involved in the regulation of the migrating motor complex (MMC) with effects on gastric emptying [131,132]. However, this group did not describe the octanoylation of ghrelin. It was soon hypothesized that ghrelin may mimic the effect of motilin on gastrointestinal motility. When ghrelin is administered iv into healthy individuals, it induces the MMC in the fasted state, inhibits gastric accommodation, and accelerates gastric emptying in the postprandial state [6,92,133,134]. Several clinical trials are currently investigating the potential of ghrelin mimetics in the treatment of hypomotility disorders (diabetic gastroparesis, postoperative ileus), but none of these has been marketed so far [135] (Table 1).

### 4.5 Rodent pharmacology studies on ghrelin's effect on food intake

Ghrelin is the only circulating hormone that, upon systemic and central administration, potently increases adiposity and food intake [2]. Similar to other GH secretagogues [136] the effect of ghrelin on adiposity is GH-independent and involves neural circuits that control food intake, energy expenditure, nutrient partitioning, and reward [3]. In the ARC, a key hypothalamic center regulating food intake and satiety [42], ghrelin increases the activity of *Npy* and *Agrp* expressing neurons while inhibiting the activity of proopiomelanocortin (*Pomc*) neurons [3]. NPY and AgRP are crucial for ghrelin's effect on feeding behavior as ghrelin fails to increase food intake in mice lacking both *Npy* and *Agrp* [137]. In line with this notion, AgRP neuron-selective GHSR re-expression in otherwise GHSR deficient mice partially restores the orexigenic response to administered ghrelin and fully restored the lowered blood glucose levels observed upon caloric restriction [138]. Of appreciable note, hyperphagia observed under pathophysiological conditions, such as streptozotocin-induced diabetes, is mediated by increased ghrelin release, which targets the ghrelin receptor on NPY and AgRP neurons [139]. A crucial regulator of *Npy* and *Agrp* expression is the hypothalamic homeobox domain transcription factor Bsx [140], which is also essential for ghrelin's ability to stimulate food intake [140].

### 4.6 Rodent pharmacology studies on ghrelin's effect on adiposity

The increased adiposity induced by the central administration of ghrelin involves the stimulation of key enzymes promoting fatty acid storage, while genes controlling the rate-limiting step in fat oxidation are decreased [141]. These actions of the brain ghrelin system on adipose tissue are mediated by the sympathetic nervous system independent of food intake or energy expenditure [141]. In addition to its actions on lipid metabolism in adipose tissue, chronic central infusion of ghrelin also increases plasma cholesterol levels, and more specifically HDL, an effect that is consistent with the fact that mice lacking both ghrelin and GHSR1a show lower plasma cholesterol levels than wild type mice [142]. Ghrelin's effects on adiposity, therefore, are achieved through centrally and peripherally mediated signaling mechanisms, including modulation of the hypothalamic melanocortinergic system and the food intake independent modulation of peripheral genetic programs regulating lipogenesis [141,143].

### 4.7 Rodent pharmacology studies using genetically engineered mouse models

#### 4.7.1 Studies in mice with adult-onset ablation of ghrelin-producing cells

The group of Joseph Goldstein and Michael Brown recently generated transgenic mice in which the diphtheria toxin receptor is expressed in ghrelin-secreting cells. Adult-onset ablation of ghrelin-producing cells in these mice, following administration of diphtheria toxin, had no effect on food intake and body weight, indicating that while ghrelin has potent pharmacological effects on food intake, energy metabolism, and body weight, it is not an essential endogenous regulator of those endpoints [144].

Despite this finding, diet-induced obesity renders NPY/AgRP neurons unresponsive to the stimulatory actions of ghrelin on food intake [143,145], an effect that is reversed with diet-restricted weight loss [146]. However, mice continue to gain adiposity on a high-fat diet (HFD) [143]; thus, reinstatement of ghrelin sensitivity with diet-induced weight loss may provide a physiological means to protect a higher body weight set point established after prolonged HFD exposure. Moreover, selective reduction of the expression of GHSR1a in the paraventricular nucleus of the hypothalamus (PVH) reduces body weight without affecting food intake [147], which supports the idea of two parallel ghrelin-responsive hypothalamic circuits that regulate food intake and adiposity independently. As yet, the differences in these neural circuits remain unknown.

#### 4.7.2 Studies in ghrelin deficient mice

In recent years, studies using genetically engineered mouse models in which the function of the endogenous ghrelin system is altered either by loss [19,148–153] or gain of function [61,154] have contributed significantly to our knowledge about the multiple facets of ghrelin action. Results from these studies must be interpreted with caution, however, as most have utilized mice of mixed background (mainly S129/C57BL6J) and S129 favors a lean phenotype [155]. In one study, ghrelin deficient mice of mixed background were reported to have lower body weight and fat mass, which might be attributed to an observed increase in energy expenditure and locomotor activity [19]. Upon HFD exposure, these mice also show a lower respiratory quotient, indicating a shift in the metabolic fuel preference toward higher lipid utilization. While these data indicate that ghrelin promotes energy conservation by increasing carbohydrate metabolism while promoting fat storage in adipose tissue, other studies using mice of mixed background show no overt changes in body weight, adiposity, or food intake between ghrelin deficient mice and wt controls under chow-fed conditions [149,150]. However, when chronically exposed to a HFD, especially beginning at an early age, these same mice show a clear metabolic benefit from ghrelin-deficiency. It should be noted that this benefit is not evident in mature, congenic knock out (ko) mice on a pure C57BL/6J background [155]. The phenotype of loss-of-function models for ghrelin depends on environmental conditions. Under sub-thermoneutral conditions accompanied by fasting, ghrelin ko mice become compromised and are unable to integrate sleep and thermoregulatory responses to metabolic challenges [156]. When chronically challenged with a HFD, ghrelin deficient mice show improved glucose disposal and insulin sensitivity compared to wt controls [19]. When crossing ghrelin deficient mice with leptin deficient ob/ob mice [157], double mutants retain the marked body adiposity phenotype of ob/ob mice. However, the double mutants show a significant decrease in basal glucose and an increase in basal insulin levels, as well as improved glucose tolerance and insulin sensitivity when stimulated with a glucose or insulin challenge, compared to native ob/ob mice. In addition, fasting glucose levels are normalized in the double mutants, compared to ob/ob mice [157].

The lack of significant changes in food intake in ghrelin deficient mice does not support a role for ghrelin as an essential ‘meal initiation’ or ‘hunger’ hormone. Nevertheless, HFD exposure of ghrelin deficient mice reveals physiological roles for ghrelin in regulating body weight and adiposity, potentially through altering fat deposition and metabolism by decreasing fuel efficiency and increasing fat oxidation.

Such improvements in body weight homeostasis might lead indirectly to improvements in glucose homeostasis; in addition, ghrelin deficiency might directly improve glucose sensitivity and pancreatic βeta cell function. The effects of ghrelin on insulin sensitivity are at least partly mediated by the central nervous system, and more specifically by AgRP neurons within the ARC. Using a tamoxifen-inducible AgRP-CreER(T2) transgenic mouse model that allows spatiotemporally-controlled re-expression of physiological levels of ghrelin receptors (GHSRs) in AgRP neurons of adult GHSR-null mice that otherwise lack GHSR expression, it was found that AgRP neuron-selective GHSR re-expression fully restored the lowered blood glucose levels observed upon caloric restriction [138]. The restoration of glucose levels was associated to glucagon rises and hepatic gluconeogenesis induction [138]

#### 4.7.3 Studies in GHSR deficient mice

The orexigenic effect of ghrelin is specifically modulated through GHSR1a, as exogenous ghrelin fails to promote food intake in mice lacking this receptor [158] and in rats treated centrally with GHSR1a antagonists [147]. Despite the well-described actions of ghrelin on NPY and AgRP neurons, very little is known about the function of other hypothalamic neuronal populations expressing the GHSR. These include the ventromedial nucleus of the hypothalamus (VMH), dorsomedial nucleus of the hypothalamus and medial preoptic area [159,160]. Elucidating the function of these populations will highlight the role of ghrelin as being more than simply a “hunger hormone.”

As in mice lacking ghrelin, mice deficient for GHSR are protected from diet-induced obesity (DIO) when fed a HFD. This might be explained, in part, by a mild hypophagia and preferential utilization of fat as an energy substrate in these mice [151,152]. Expression of *GHSR* antisense RNA under the TH promoter in the ARC of rats results in hypophagia and decreased body weight and body fat [161]. Compared to wt littermate controls, *GHSR* deficient mice also show improved glucose disposal and insulin sensitivity upon HFD exposure. However, body weight and fat mass are not affected when male *GHSR* deficient mice are maintained on a standard chow diet [151,158]. Consistently, ablation of ghrelin receptor reduces adiposity and improves insulin sensitivity during aging by regulating fat metabolism in white and brown adipose tissues [25]. The effects of ghrelin on glucose metabolism during aging might be associated to GH levels, as it is known that circulating GH levels, which cause insulin resistance [162], are decreased in later stages.

Interestingly, simultaneous deletion of both ghrelin and *GHSR* results in lower body weight and fat mass even when the double mutant mice are fed a standard chow diet [153]. One possible explanation for this finding is the potential existence of additional ligands for the GHSR and/or additional receptors for ghrelin, which may exacerbate the metabolic phenotype of double mutant mice where ghrelin and GHSR are inactivated [153].

Another possibility is that GHSR may affect food intake independently of ghrelin signaling, e.g. by heterodimerization with other receptors such as the dopamine receptor [163] or GPR83 [164]. Of note, the impact of GHSR signaling on food intake, body weight, or energy and glucose homeostasis might be influenced by the receptor's intrinsic constitutive activity [165], thus complicating the direct comparison of metabolic phenotypes of ghrelin and GHSR deficient mouse models. However, given the level of GHSR1a expression found in native tissues, it is doubtful that basal activity is a contributing factor. Furthermore, this interpretation might be confounded by the observation that GHSR1a, but not ghrelin, is essential for appetite regulation by dopamine receptor subtype-2 [166].

#### 4.7.4 Studies in GOAT deficient mice

Generation of mice with the genetic inactivation of GOAT also has proven to be a useful tool to assess the role of des-acyl ghrelin without *in vivo* octanoylation [20,61,167]. Work by the laboratories of Joseph Goldstein and Michael Brown in young fat-depleted *GOAT* ko mice showed that acyl-ghrelin is of crucial importance for preventing life-threatening events of hypoglycemia under conditions of acute caloric restriction, an effect attributable to ghrelin's ability to promote the release of GH from the anterior pituitary [20,168,169]. Accordingly, when body fat is reduced by caloric restriction, ghrelin stimulates GH secretion, which allows maintenance of glucose production, even when food intake is eliminated. In line with this role of ghrelin to prevent hypoglycemia, adult-onset ablation of ghrelin producing cells induces profound hypoglycemia during prolonged caloric restriction [144]. Moreover, severe caloric restriction substantially increases plasma GH levels and promotes hepatic autophagy in wt mice, allowing the mice to maintain viable levels of blood glucose while lethal hypoglycemia and a blunted GH increase is observed in mice deficient for GOAT [170]. Hypoglycemia is also observed in GHSR-null mice following the same prolonged caloric restriction protocol [138] and upon initiation of acute caloric restriction of both ghrelin ko and *GHSR* ko mice, although both genotypes adapted after 14 days [155]. Also pharmacological inhibition of GOAT has been shown to improve glucose disposal and to enhance insulin secretion, an effect notably not seen in *GHSR* ko mice [60].

### 4.8 FTO and ghrelin

Several SNPs within the first intron of the fat mass and obesity-associated gene (*FTO)* are robustly associated with increased BMI and adiposity across different ages and populations [171–176]. Subjects homozygous for the obesity-risk (A) allele of SNP rs9939609 have a 1.7-fold increased risk for obesity and exhibit overall increased *ad libitum* food-intake [177–179], particularly fat consumption [177,179–181], and impaired satiety [182,183] compared to subjects homozygous for the low-risk (T) allele. Recently, a series of studies implicated ghrelin in mediating this altered feeding behavior. In two independent cohorts of normal-weight, adiposity-matched individuals with either *FTO* rs9939609 TT or the obesity risk AA genotype [184], AA subjects exhibited attenuated post-meal suppression of both hunger and circulating acyl-ghrelin levels. Using fMRI, these studies demonstrated that *FTO* rs9939609 genotype modulated the neural responses to food images in homeostatic and reward brain regions. Furthermore, AA and TT subjects exhibited divergent neural responsiveness to circulating acyl-ghrelin within brain regions that regulate appetite, reward-processing and incentive motivation. At the molecular level, FTO directly demethylates *N*^*6*^-methyladenosine (m^6^A), a naturally occurring adenosine modification in RNA and *ghrelin* mRNA has been identified as an FTO target [184,185]. *FTO* over-expression in MGN3-1 cells, a validated ghrelin cell line, reduced ghrelin mRNA m^6^A methylation, increased *ghrelin* mRNA abundance and the synthesis and secretion of acyl-ghrelin. Furthermore, subjects with the A allele of rs9939609 exhibit increased *FTO* expression [184,186] and decreased *ghrelin* m^6^A methylation coupled with increased *ghrelin* expression [184]. Interestingly, FTO also regulates the m^6^A methylation and expression of key molecular components of the mid-brain dopaminergic system, which is known to play a key role in mediating the rewarding effects of ghrelin [187]. Altered dopaminergic signaling may account for the altered neural ghrelin sensitivity reported in rs9939609 *FTO* AA subjects [184]. This suggests that the known actions of acyl-ghrelin, increased food intake, increased adiposity, preference for high-fat food, enhanced operant responding for food rewards, induced conditioned place preference for food rewards and a role in cue-potentiated feeding are strikingly similar to the feeding phenotype of rs9939609 AA subjects. However, while these findings of altered ghrelin function in *FTO* rs9939609 AA subjects provide a parsimonious explanation for the obesity risk phenotype seen in these subjects, given the pleiotropic effects of FTO a number of other mechanisms could also be implicated.

### 4.9 Additional functions of ghrelin

In addition to ghrelin's role in glucose and energy homeostasis, research over the last 15 years has revealed a surprising variety of additional physiological functions of ghrelin in areas as distinct as learning and memory [188–190], psychological stress, mood and anxiety [191,192], depression [26,193,194], thymopoiesis [195], sleep/wake rhythm [7–9,196,197], and aging [198,199]. Recent pharmacological intervention trials also point to a neuroprotective role of ghrelin in neurodegenerative diseases (e.g., Parkinson's disease) [198,199]. The ghrelin system's neuroprotective effects are apparent in mouse models of chronic psychosocial stress, wherein stress-induced decreases in adult hippocampal neurogenesis become exaggerated in mice lacking GHSRs [200]. Additional studies examining the genetic and pharmacological modulation of the ghrelin system will help elucidate these novel roles of ghrelin.

## 5 Ghrelin action in the brain

### 5.1 Hypothalamic effects of ghrelin on energy metabolism

Although an important site of action of ghrelin on the control of food intake is the ARC, ghrelin administration into other hypothalamic sites, including the PVH [201,202] and the lateral hypothalamus [203] also promote a positive energy balance. In the hypothalamus, ghrelin triggers endocannabinoid release [204], leading to activation of the calcium/calmodulin-dependent protein kinase 2 (CaMKK2) and increased phosphorylation of the energy sensor AMP-activated protein kinase (AMPK) [205–207]. Ghrelin mediated activation of GHSR1a also triggers hypothalamic sirtuin 1 (Sirt1) [208,209], which deacetylates p53, leading to increased phosphorylated levels of AMP-activated protein kinase (AMPK) [206] and to the inactivation of enzymatic steps of *de novo* fatty acid biosynthetic pathway in the VMH [207]. These molecular events induce changes in uncoupling protein 2 (UCP2) [210] and the upregulation of the transcription factors Bsx [140], forkhead box O1 (FoxO1), and cAMP response-element binding protein (pCREB) [211] followed by subsequent activation of downstream signaling pathways. Within the hypothalamus, ghrelin increases expression of the prolyl carboxypeptidase (PRCP) a negative regulator of the melanocortin 4 receptor agonist α-melanocyte stimulating hormone (α-MSH) [212] and the mechanistic target of rapamycin (mTOR) in the ARC. In fact, central inhibition of mTOR signaling with rapamycin decreases ghrelin's orexigenic action [213].

### 5.2 Non-hypothalamic effects of ghrelin on energy metabolism

Ghrelin also promotes a positive energy balance when administered to non-hypothalamic sites such as the hindbrain [214–216] and limbic/paralimbic regions including the amygdala [202,217,218]. A recent study employed a genetically-engineered mouse model with *Ghsr* expression limited to the hindbrain to determine if such site-selective, hindbrain GHSR expression is sufficient to mediate ghrelin's actions on food intake and blood glucose [219]. When these animals were provided food *ad libitum*, hindbrain-specific GHSR expression was not sufficient to permit the characteristic orexigenic response to subcutaneous ghrelin administration that is observed in wt animals. With respect to the modulation of glucose homeostasis, hindbrain GHSR expression was sufficient to defend against the exacerbated fasting-induced fall in blood glucose that is otherwise observed in mice with global GHSR deficiency. These data help clarify the relevant sites of ghrelin receptor action in the brain in the modulation of food intake and blood glucose and complement a prior study investigating the effects of tyrosine hydroxylase-Cre-driven GHSR expression, in which GHSR expression occurs selectively in catecholaminergic (predominantly dopaminergic) neurons, such as those in the VTA [28]. Notably, and unlike with paired mesoderm homeobox 2B (Phox2b) cre-driven hindbrain *GHSR* expression, catecholaminergic *GHSR* expression is sufficient to partially rescue ghrelin-stimulated acute food intake, while fully restoring the ability of administered ghrelin and chronic stress to modulate food reward [28]. Also, unlike with the hindbrain-selective *GHSR* expression, fasting blood glucose levels are not rescued by selective *GHSR* expression in catecholaminergic cells [28].

Several lines of evidence indicate that the brainstem contributes to ghrelin's orexigenic action, as peripheral administration of GHSs and intracerebroventricular (icv) ghrelin administration increase c-Fos expression in the nucleus tractus solitaris and the area postrema [220,221]. The role of the vagus nerve in the regulation of ghrelin-induced food intake is more controversial, however, as one study shows that blockade of gastric vagal afferents diminishes ghrelin's effect on food intake and decreases ghrelin induced c-Fos expression in the ARC [222], while another study reports that gut vagal afferents are not necessary for the hyperphagic action of ghrelin [223]. As gastrectomy is accompanied by vagotomy, the fact that ghrelin analogs are anabolic when given after gastrectomy suggests that the vagus is not essential for ghrelin's orexigenic effects [224,225].

### 5.3 Ghrelin and the reward system

Ghrelin engages reward neurocircuits that are activated by drugs of abuse [15,226–232]. In particular, the central ghrelin signaling system seems to be important for the rewarding properties of alcohol [228], nicotine [233,234] and cocaine [235]. One of the neurocircuits involved in these effects is the mesolimbic, dopaminergic pathway that projects from the VTA to the nucleus accumbens (NAc) [229,236], a pathway with a key role in reward-seeking behavior. Acting on this pathway, ghrelin affects the motivation and drive to eat. Ghrelin administration to the VTA and the NAc increases both food intake [232,237] and extracellular dopamine [13,238]. Underscoring the importance of dopamine for ghrelin's orexigenic effects is the finding that intra-VTA delivery of a ghrelin antagonist blocks the ability of parenteral ghrelin to increase feeding [239]. Dopamine modulates the incentive salience of food [240] and the animal's willingness to work for food [241]. In short, it increases feeding by increasing the drive, arousal, foraging, and motor hyperactivity that occur during food anticipation. For example, ghrelin ko mice do not show the normal anticipatory locomotion to scheduled meals [242,243]. Ghrelin also has direct effects on two other regions implicated in the control of feeding: the hippocampus and amygdala, where it facilitates learning and memory [188] and emotional arousal [244] and cue-potentiated feeding [189].

GHSR1a and dopamine receptor-2 (D2R) are present as GHSR1a:D2R heterodimers in native hypothalamic neurons and the inhibitory effects of D2R signaling on food intake is dependent on the presence of GHSR1a. *GHSR* ko mice and wt mice treated with a selective GHSR1a antagonist are resistant to the anorexigenic effects of a DRD2 agonist. Remarkably, ghrelin ko mice are fully sensitive to DRD2 agonist suppression of food intake, demonstrating a dependence on GHSR1a but not on ghrelin [163]. At the level of the VTA, but not the NAc, ghrelin increases motivation for food, reflected by an increased lever pressing for sucrose pellets in a progressive ratio task [231]. Interestingly, the VTA-driven effects of ghrelin on food motivation involve different neurocircuits than those involved in food intake. NAc delivery of dopamine receptor (D1R and D2R) antagonists blocked the effects of intra-VTA infused ghrelin on food motivation/reward behavior but not food intake, suggesting that the VTA-NAc dopamine reward pathway is important for food motivation but not food intake [245]. Given that central blockade or stimulation of the dopamine receptors 1, 2, and 3 suppress the effects of icv delivered ghrelin on food intake [246], it can be inferred that dopamine has a role outside of this classic reward pathway to regulate ghrelin's orexigenic effects. Consistent with this, GHSR1a and D2R have been shown to interact within hypothalamic neurons blunting the anorexigenic actions of D2R agonism [163]. Divergence in the mesolimbic circuitry mediating ghrelin's orexigenic versus and food reward effects also occur at the VTA level and can be parsed using opioid and NPY Y1 receptor antagonists [232,236]. Interactions between ghrelin and the opioid system occur not only in the mesolimbic dopamine system but also in the hypothalamus. More precisely, GHSR1a and kappa opioid receptor colocalize in hypothalamic areas and the blockade of the kappa opioid receptor in the ARC is sufficient to blunt ghrelin-induced food intake [247]. Collectively, studies linking ghrelin to the mesolimbic reward circuitry suggest that ghrelin's role in hunger and meal initiation may extend to reward-driven behaviors, including food motivation.

### 5.4 The role of ghrelin learning and memory performance

Ghrelin exhibits dense receptor expression in the hippocampus [188], where it has been found to forms of learning and memory performance in rodents. For example, ghrelin administration has been shown to promote long term potentiation in the hippocampus, increase spine density of neurons in the hippocampal CA1 region, and enhance performance in several types of hippocampal-dependent learning and memory tasks [188,248]. Additionally, ghrelin has been shown to increase survival and reduce cell death of hippocampal neurons following ischemia/reperfusion injury [249]. Finally, it was recently shown that ghrelin cells receive direct synaptic input from the suprachiasmatic nucleus and the lateral geniculate nucleus, suggesting that ghrelin is implicated in mediating circadian and visual cues for the hypothalamic arousal system [250]

## 6 Regulation of ghrelin secretion

Despite a growing body of literature characterizing ghrelin action and the distribution of ghrelin cells, relatively little is known about the exact molecular pathways responsible for the biosynthesis and release of ghrelin. Instead, most of what is known regarding the control of circulating ghrelin is on a broader, systemic level.

### 6.1 Ghrelin secretion in response to fasting and feeding

It has been known for several years that ghrelin levels rise pre-prandially and decrease to baseline levels within the first hour after a meal [37], a pattern that can be entrained by artificial meal schedules [251]. The magnitude of ghrelin reduction is proportional to the caloric load and macronutrient content, and ingested lipids are the least effective suppressor of plasma ghrelin [252]. Also, it is well established that plasma levels of both acyl and des-acyl ghrelin rise with prolonged food deprivation, increases that can be blocked by reserpine, which depletes adrenergic neurotransmitters from sympathetic neurons [253]. Sham feeding also suppresses ghrelin levels [254]. Furthermore, the recovery of ghrelin levels does not seem to be an important determinant of intermeal intervals [255], and mice that lack ghrelin have normal meal intervals.

### 6.2 Ghrelin levels in pathological conditions

It is well established that ghrelin plays a role in long-term energy balance regulation, defending against prolonged energy deficiency. Accordingly, in humans, circulating ghrelin levels are generally inversely associated with weight gain, adiposity, and insulin resistance [256] and positively correlated with weight loss induced by exercise, low-calorie diet, mixed life-style modification, anorexia nervosa and cachexia due to chronic obstructive lung disease (COPD) or chronic heart failure (CHF) [71,257,258]. Ghrelin levels are low in obesity [259] and even lower in obese binge eaters [260], suggesting that, in these instances, ghrelin is a consequence rather than a cause of overeating. In line with the observation that ghrelin levels increase by fasting, plasma levels of ghrelin are high in patients with cachexia or in patients with eating disorders such as anorexia nervosa and bulimia nervosa [as reviewed in [166,258,261]]. Interestingly, extreme fasting reduces ghrelin levels in healthy subjects [262–264]. These effects are prevented by subdiaphragmatic vagotomy and, separately, by administration of the anticholinergic agent, atropine [265]. Conversely, obese patients with Prader-Willi syndrome are hyperphagic and have very high circulating ghrelin levels [266,267]. Elevated levels of ghrelin are further reported from patients with Hashimoto's Thyroiditis [268] but not from overweight/obese patients with Bardet-Biedl syndrome [269], Cushing's Disease [270,271], or HIV-Lipodystrophy [272]. Taken together, these data suggest that changes in circulating plasma levels of ghrelin may be relevant for the increase in adiposity in humans, although the degree of its contribution remains to be determined.

### 6.3 Ghrelin levels after bariatric surgery

Recent data demonstrate variable effects of various bariatric surgery procedures (i.e. Roux-en-Y gastric bypass, vertical sleeve gastrectomy, laparascopic adjustable banding) on ghrelin levels (generally demonstrating decreases post-surgery) [273–279], shedding light on how ghrelin exerts its mechanistic effects in the gastrointestinal tract (reviewed in [280]). Low ghrelin levels have been reported for individuals after weight loss induced by Roux-en-Y gastric bypass, initially believed to play a key role in the decreased appetite observed after this surgery [257]. Subsequent data, however, show that ghrelin levels rise within the first year after this surgery in humans [281] and within 6 weeks after surgery in mice [282]. Additionally, compared to wt control mice, in ghrelin ko mice, vertical sleeve gastrectomy is equally efficient in lowering body weight [283], indicating a ghrelin independent effect in this type of bariatric surgery.

### 6.4 Ghrelin secretion in response to external food cues

Ghrelin release during fasting is mediated via activation of the autonomic nervous system. There is evidence that both cholinergic and adrenergic neurotransmission are involved in the release of ghrelin. Stimulation of ghrelin release in response to cholinergic activation by pharmacological substances or sham-feeding has been reported in humans and rodents [284–286]. In addition, food deprivation-induced elevation of plasma ghrelin levels is driven by an increased vagal efferent tone [284,285]. However, these results are not confirmed by *in vitro* studies either in a ghrelinoma cell line [287] or primary cell culture from rat stomach [288].

External food cues such as sight, smell, and taste trigger the cephalic phase of ingestive behavior, which consists of increased gut motility, gut hormone secretion, and autonomic arousal [289]. This response, in turn, triggers central arousal and incentive mechanisms that promote food consumption. The cephalic response includes ghrelin release, which increases after exposure to food cues in humans [290]. Conversely, recent evidence suggests that anticipation of the caloric content of an investigator-supplied milkshake modulates the post-prandial reduction in ghrelin levels [291]. When subjects believed they were consuming a high calorie rather than a “healthy” milkshake, their ghrelin levels were much more reduced. In sum, ghrelin secretion is part of a CNS-gut control loop for feeding; food cues promote ghrelin release from the stomach, which feeds back to the CNS to activate hypothalamic and dopaminergic feeding centers. This feedback allows other factors such as chronic stress, negative energy balance, leptin and insulin to affect motivation to feed by enhancing or reducing the cephalic release of ghrelin [290,292].

### 6.5 Suppression of ghrelin secretion

There has been significant interest in unveiling the mechanisms involved in postprandial suppression of circulating ghrelin levels. The placement of a pyloric cuff in rats to block normal flow of gastric contents into the duodenum prevents drops in circulating ghrelin usually observed following intragastric infusion of glucose [293]. Furthermore, stomach distention by infusion of water into animals whose gastric outflow was occluded at the level of the pylorus also was ineffective in changing ghrelin levels [294]. Thus, it appears that neither nutrient detection by the stomach nor gastric distention is sufficient for eliciting the usual postprandial fall in circulating ghrelin. On the other hand, both intraduodenal and intrajejunal administration of nutrients via intestinal cannulas lower circulating ghrelin levels.

Within the stomach, considered the predominant source of circulating ghrelin, ghrelin cells tend to cluster towards the base of the gastric mucosal glands and are of the round, closed-type variety that do not have direct contact with gastric luminal contents (reviewed in [295]). There is also credible evidence that ghrelin cells exist, although in fewer numbers, throughout the entirety of the gastrointestinal tract, including the duodenum, where more elongated, opened-type ghrelin cells, which have direct contact with the intestinal lumen and may be regulated differently than their gastric counterparts.

### 6.6 Ghrelin secretion in response to dietary macronutrients

A recent randomized, within-subjects crossover human trial helped characterize the manner in which different types of nutrients influence the pattern of postprandial fluctuations in plasma ghrelin levels [296]. For this study, isocaloric, isovolemic beverages, composed primarily of carbohydrates, proteins, or lipids, were administered to volunteers whose plasma levels of acyl and total ghrelin were measured multiple times over the next 6 h. The lipid drink was the least effective, and the protein drink was most effective in lowering ghrelin levels. Although the carbohydrate drink resulted in the largest drop in ghrelin initially, it was the only drink to induce a subsequent rebound to above pre-prandial levels. Interestingly, ghrelin levels are also suppressed by sham feeding in humans [254], underscoring the role of the cephalic phase in the modification of ghrelin levels prior to and in response to a meal.

Animal data suggest that these nutrient-associated decreases in circulating plasma ghrelin levels do not appear to involve the vagus nerve [223], which relays interoceptive sensory information from the viscera to the CNS and helps control visceral function. In fact, following intragastric gavage of a liquid diet to animals that had undergone subdiaphragmatic vagotomy, decreased ghrelin levels were observed [265]. However, the vagus nerve does seem to play a role in the rise of plasma ghrelin associated with a negative energy balance, as 48 h food deprivation-associated elevations in circulating ghrelin are prevented by subdiaphragmatic vagotomy or administration of atropine [265].

### 6.7 Hormones and neurotransmitter regulating ghrelin secretion

Recent studies have demonstrated that increases in ghrelin levels also occur upon acute or chronic stress that is not necessarily related to negative energy balance [reviewed in [26,28,297,298]]. Indeed, sympathetic activation increases ghrelin secretion [192]. Both *in vitro* and *in vivo* studies have demonstrated release of ghrelin in response to sympathetic stimulation mediated viaβ1-adrenergic receptors present on the ghrelin cell [253,284,287,288,299].

To more directly study the determinants of ghrelin secretion, a recent study performed local infusion of candidate compounds into the gastric submucosa followed by measurement of ghrelin mobilization via implanted microdialysis probes [300]. Using this method, epinephrine, norepinephrine, endothelin, and secretin were found to stimulate ghrelin release. In contrast to the stimulation of ghrelin release by activation of the sympathetic nervous system (SNS), the inhibition of ghrelin release seems primarily mediated by gastrointestinal hormones released during nutrient digestion, such as somatostatin and gastrin releasing peptide/bombesin. Numerous other hormones as well as many neurotransmitters, neuropeptides, glucose, and amino acids had no effect. Although this microdialysis technique and others, such as an *ex vivo* stomach explant culture system [294,301], help focus on locally acting compounds that influence ghrelin release, the techniques do not discriminate between compounds that act directly on ghrelin cells versus those that act indirectly via effects on neighboring cells. In summary, the regulation of ghrelin release is a complex process that is tightly controlled by both, the SNS and the gastrointestinal tract and which involves hormonal stimuli not necessarily involved in energy balance regulation. The observation that also factors not involved in systems metabolism regulate ghrelin secretion speaks for a broader physiological role of ghrelin and is in line with a multitude of ghrelin effects beyond the regulation of hunger and satiety. A potential beneficial effect of this complexity is the possibility to target the ghrelin system for the treatment of pathological conditions not necessarily related to a negative energy balance, such as e.g. gastroparesis.

Recent studies showed that that the ghrelin cell is chemosensory and contains taste receptors similar to those located in the tongue. Indeed, the ghrelin cell is co-localized with the gustatory G-proteins, α-gustducin and α-transducin. Studies in α-gustducin ko mice show that α-gustducin partially mediates the effect of bitter tastants on ghrelin release [302]. Similarly, the taste 1 receptor subtype, TAS1R3, involved in sensing both sugars and amino acids, is co-localized with ghrelin cells in the antrum [303]. The closed-type ghrelin cells in the stomach may receive chemosensory input from the bloodstream while the opened-type cells in the duodenum may respond to luminal stimuli. The long-chain fatty acid sensing receptor GPR120 is co-localized with ghrelin containing cells in the duodenum but not in the stomach and has been shown to play a role in the lipid-sensing cascade of the ghrelin cell [68]. Free fatty acid receptor 1 (FFAR1), involved in sensing of long/medium chain fatty acids, is expressed only in the des-acyl (non-active)-containing ghrelin cell population in the stomach, and its function is unclear [68]. More studies are warranted to elucidate the role of taste receptors in the effects of nutrients on ghrelin secretion.

It is likely that recent findings and new tools will provide greater insight into the regulation of ghrelin secretion (as reviewed in [304]). For instance, the development of novel, high-throughput sandwich enzyme linked immunosorbent assays or radioimmunoassays for the specific and sensitive detection of acyl-ghrelin as well as new mass spectrometry methods will permit accurate means to detect the different forms of circulating ghrelin and determine how various manipulations influence the levels of these different forms [305].

Another key development is the identification of GOAT, the enzyme responsible for catalyzing ghrelin's unique post-translational modification [56,57]. Recent work involving genetic manipulations of *GOAT* expression and the aforementioned mass spectrometry methods are challenging some of the accepted dogma about ghrelin secretion and regulation and suggest that ghrelin acylation and the secretion of acylated ghrelin represent two independent processes [61].

### 6.8 Ghrelin secretion regulated by G-protein coupled receptors

**Figure 4:**
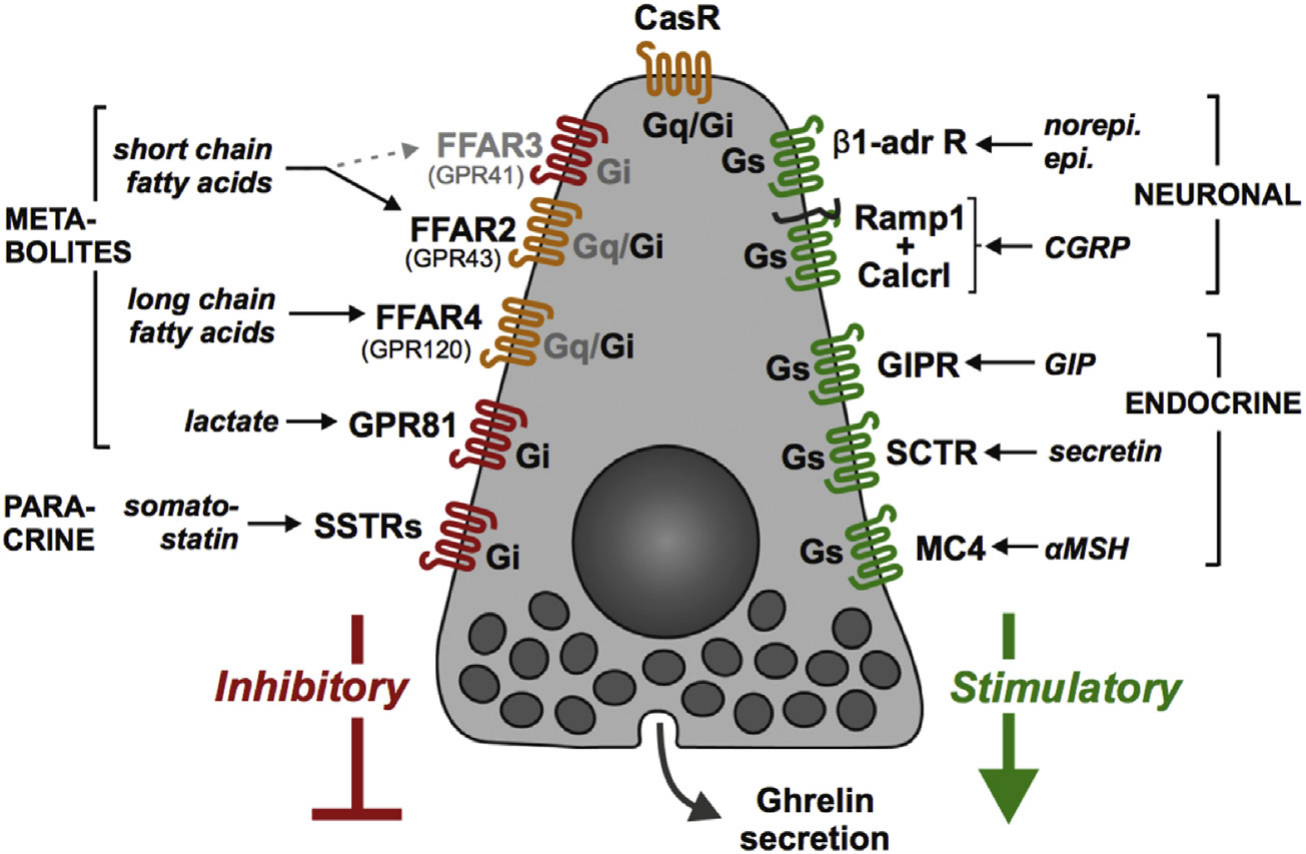
Schematic overview of the 7TM receptors judged to be either stimulating (in green to the right) or inhibiting (red or orange to the left and top) ghrelin secretion directly on the ghrelin cell. The main signaling pathway (Gαs or Gαi) employed by each of the receptors in the ghrelin cell is indicated inside the receptor in black. Figure taken from Engelstoft et al., Mol Metab. 2013 [303].

Over the last few years, several new ghrelin secretion models have been developed. Quantitative PCR analysis of FACS-purified gastric ghrelin cells identified a series of G-protein coupled receptors (GPCRs) that regulate ghrelin secretion. The GPCRs stimulating ghrelin secretion were mainly Gs-coupled and include the β1-adrenergic receptor, the GIP receptor, the secretin receptor (SCTR) and, interestingly, the sensory neuropeptide receptor CGRP, and the melanocortin 4 receptor (MC4R) [306]. GPCRs inhibiting ghrelin release were Gq and/or Gi coupled and included the somatostatin receptors (SSTRs), the lactate receptor (GPR81) and receptors for short chain fatty acids (FFAR2) and long chain fatty acids (FFAR4) (Figure 4) [306].

### 6.9 Models to assess ghrelin secretion using transgenic mice

Other models developed to assess ghrelin secretion include genetically-engineered mouse models in which green fluorescence protein reports on the location of ghrelin-expressing cells. This method enables direct visualization of ghrelin cells, and fluorescence activated cell sorting-mediated isolation of ghrelin cells for expression analyses and cell culture [295,307,308]. Primary cell cultures of dispersed gastric mucosal cells from adult mice and 8-day-old rat pups also have been developed to investigate ghrelin secretion [288,307,309]. Ghrelin-secreting immortalized cell lines developed from ghrelinomas in the stomachs (SG-1, MGN3-1) and pancreatic islets (PG-1) of transgenic mice expressing SV40 large T-antigen under the control of preproghrelin promoter are now available [253,310]. These ghrelinoma cell lines retain many of the key, phenotypic features of ghrelin cells and respond to many of the same regulators of ghrelin secretion that have been described *in vivo* and in primary culture systems [253,288,307,310].

Using these models, the modulation of ghrelin release by peptide hormones, monoaminergic neurotransmitters, glucose, fatty acids, second messengers, potential downstream effector enzymes, and channels has now been investigated. Insulin, glucagon, oxytocin, somatostatin, dopamine, glucose, and long-chain fatty acids all have been shown to regulate ghrelin secretion through their direct interaction with ghrelin cells [253,288,307–310]. In addition, all of these models, as well as related *in vivo* studies, have been used to confirm that the catecholamines norepinephrine and epinephrine act as direct ghrelin secretagogues [253,287,288,299,307]. These data are supported by high levels of β1-adrenergic receptor expression in ghrelin cells enriched from the stomach of ghrelin-green fluorescent protein reporter mice as well as in the SG-1 and PG-1 ghrelin cell lines [253]. Forskolin, a potent activator of adenlyl cyclase, mimics the effect of norepinephrine [253], suggesting that activation of adenylyl cyclase and an ensuing elevation of cAMP occurs following engagement of β1-adrenergic receptors, as has been shown in other cell systems [311,312]. Interestingly, neuronal and endocrine signals have stimulatory effects on ghrelin secretion whereas paracrine signals and macronutrient metabolites such as fatty acids inhibit ghrelin release [306].

Altogether, these findings, along with the aforementioned microdialysis experiments, link fasting-induced stimulation of the sympathetic nervous system and ensuing release of norepinephrine locally in the stomach wall to the release of ghrelin [168,299]. New transgenic and cell culture models should allow for many more discoveries in the regulation of ghrelin secretion and other aspects of ghrelin cell physiology. In summary, recent studies reveal a comprehensive picture of the receptor repertoire expressed on the ghrelin cells, which allows for deeper analyses of the physiological properties and pharmacological potential of the ghrelin cell.

## 7 Open questions

### 7.1 What is the role of des-acyl ghrelin?

#### 7.1.1 Enzymes regulating des-acylation of ghrelin

Depending on the species, the serum half-life of acyl-ghrelin varies between 240 min in humans and 30 min in rats [313]. The differences in ghrelin's half-life might be explained by the fact that the enzymes responsible for the des-acylation and cleavage of ghrelin differ remarkably across species. Butyrylcholinesterase is the predominant enzyme responsible for ghrelin inactivation in humans whereas carboxylesterases allow for an eight times faster ghrelin des-octanoylation in rodents [313]. In rodents, des-octanoyl ghrelin is localized in two ghrelin cell populations in the stomach: cells that contain only des-octanoyl ghrelin and cells that contain both des-octanoyl and octanoyl ghrelin [302,314]. Most studies have analyzed both des-acyl and acyl ghrelin finding that the majority of ghrelin in circulation is des-acylated. No receptor for des-acyl ghrelin has been identified and a recent study in which acyl ghrelin was assessed in human plasma using mass spectrometry suggests that all ghrelin in circulation is acylated and that des-acyl ghrelin may be an artifact of sample handling [315].

#### 7.1.2 Effects of des-acyl ghrelin

Nevertheless, several studies suggest that des-acyl ghrelin promotes differentiation and fusion in C2C12 skeletal muscle cells [29], prevents muscle atrophy [30], elicits GHSR1 independent effects on energy and glucose metabolism [316–318], and exerts a cardioprotective effect on endothelial cells and cardiomyocytes [319,320]. Central infusion of des-acyl ghrelin into the third ventricle of rats increases short-term food intake, whereas peripheral administration of des-acyl ghrelin seems to have no direct effect on food intake [317]. The effect of des-acyl ghrelin on food intake is independent of GHSR1a and Npy signaling and might be orexin mediated [317]. Other studies, however, suggest that des-acyl ghrelin decreases food intake in rats and disrupts stomach motor activity under conditions of fasting [321]. Transgenic mice overexpressing des-acyl ghrelin have reduced body fat mass when fed a regular chow diet and are protected from diet-induced obesity when challenged with a HFD [321]. Although there are speculations about a potential receptor for des-acyl ghrelin located in the cardiovascular system [320], to date no such receptor has been identified.

Growing evidence points to a GHSR1a-independent role of des-acyl ghrelin in glucose metabolism, possibly antagonizing the effect of acyl-ghrelin. The often reported increase of plasma glucose levels and decrease of plasma insulin levels upon ghrelin administration [84–86,88,89] seem to be antagonized by co-administration of des-acyl ghrelin [322]. Several human studies report a positive relationship between des-acyl ghrelin and insulin sensitivity [323,324], although other studies do not support this finding [325]. The effect of des-acyl ghrelin on glucose metabolism might be triggered indirectly via modulation of lipid metabolism, as transgenic mice overexpressing des-acyl ghrelin have lower body fat mass, lower body weight gain, and improved insulin sensitivity compared to wt controls [318,326]. Furthermore, administration of des-acyl ghrelin decreases activation of gene programs regulating lipogenesis [327]. A more recent study in mice shows that chronic, subcutaneous administration of des-acyl ghrelin prevents the typical metabolic alterations caused by chronic HFD exposure, such as increased expression of pro-inflammatory cytokines and the development of HFD-induced glucose intolerance and insulin resistance [328]. Conversely, mice overexpressing ghrelin driven by the neuron-specific enolase (NSE) promoter develop age-related glucose intolerance despite having lower body weight [18]. Recent observations suggest a direct role on glucose metabolism based on observations indicating that des-acyl ghrelin promotes survival of pancreatic β-cells and as des-acyl ghrelin prevents the diabetogenic effect of streptozotocin [329–332]. Other data suggest that des-acyl ghrelin administered centrally to mice at high pharmacological doses acts to increase adiposity and glucose-stimulated plasma insulin through a GHSR-dependent mechanism [333].

### 7.2 Is ghrelin a ‘hunger’ hormone?

Ghrelin levels rise pre-prandially, and administered ghrelin reliably increases food intake in humans and rodents [2,94], supporting a role for ghrelin in hunger, meal initiation, and feeding behavior in normal physiology. The acute, orexigenic effects of ghrelin, however, are most profound when ghrelin is delivered centrally, perhaps reflecting more widespread and simultaneous activation of diverse CNS sites. Indeed, orexigenic effects are observed not only after parenchymal delivery to the hypothalamic, brainstem and mesolimbic reward areas but also when administered to brain areas with a less well-established role in feeding control such as the amygdala [217], hippocampus [189] and dorsal raphe nucleus [334]. Recruitment of diverse feeding pathways by endogenous ghrelin may be under physiological control, perhaps reflecting food availability and/or nutritional status. Consistent with this, the ARC appears to show increased responsiveness to ghrelin in fasted rats relative to fed rats [335], and more ghrelin appears to gain CNS access in the fasted state [336].

It is not at all clear that ghrelin's acute orexigenic and chronic pro-obesity effects are coupled. While ghrelin may provide an acute hunger signal in the pre-prandial period, there is little evidence to suggest that sustaining high ghrelin levels induces hyperphagia in the long term. Despite numerous studies showing increased food intake upon acute or chronic systemic ghrelin application, mice deficient for ghrelin, *GHSR* or *GOAT*, nor transgenic mice overexpressing ghrelin and/or *GOAT* show alterations in food intake compared to wt controls [61,149,153]. Also, ghrelin antagonists, which were developed as anti-obesity drugs, do not appear to have chronic anorexigenic properties *per se* [337]. It may be helpful, therefore, to separate the suggested role for ghrelin as a hunger-promoting hormone in normal physiology from its therapeutically relevant, long-term obesogenic effects, which may be less linked to feeding control.

The surge in ghrelin before a meal could be linked to another role for ghrelin – to prepare the organism for incoming food in order to metabolize and store energy efficiently [61]. In line with this, ghrelin activation is influenced greatly by dietary lipids [61,63], and ghrelin might signal to the brain that abundant calories are available to acutely fill the organism's fuel stores.

One might argue that the typical increase of plasma ghrelin levels during prolonged food deprivation and the increase of ghrelin before a meal followed by the subsequent decrease afterwards clearly point to its role as a ‘hunger’ hormone. However, as discussed, the surges in ghrelin before a meal could also be explained by the theory that ghrelin prepares the organism for incoming food in order to metabolize and store energy efficiently [61]. In line with this, ghrelin activation is highly influenced by dietary lipids [61,63], and, therefore, ghrelin might signal to the brain that abundant calories are available to acutely fill the organism's fuel stores. The observation that ghrelin, independent of its effect on food intake, stimulates genetic programs regulating lipogenesis [141,143] is in line with this proposed role as a lipid sensor (as discussed in section 3.3).

### 7.3 Is the ghrelin receptor still a druggable target?

Ghrelin and its agonists appeal to those who desire to exploit the potent anabolic biology of the hormone. Such attention is directed at cachexic and frail states. Ghrelin has a very short half-life but the peptide can be engineered for a more sustained delivery and better pharmacokinetic properties. Treatment with ghrelin by infusion may be indicated in very acute circumstances when a short-term anabolic state is desired, such as prior to an elective surgery. The non-peptide GHSR1a agonists developed prior to the discovery of ghrelin are orally bioavailable; most importantly, they produce significant exposure levels for up to 24 h. The extended half-life of these compounds is of metabolic and functional importance, because, in contrast to ghrelin, chronic administration of non-peptide agonists results in sustained but modest increases in GH and IGF-1 superimposed on endogenous GH/IGF-1 without an increase in cortisol [338]. Interestingly, in obese subjects the anabolic effects of MK0677 produce an increase in the ratio of lean/fat mass [339]. Chronic therapy with orally active stable GHSR1a agonists may have utility in frail, elderly subjects because MK0677 rejuvenates the GH/IGF1 axis by enhancing pulse amplitude of episodic GH release to match the physiological profile of young adults [338]. This effect is consistent with rescue of the epigenetic-mediated age-dependent decline in *GHSR* expression that reduces ghrelin sensitivity [340]. Indeed, encouraging results were observed in elderly patients recovering from hip fracture [341], with beneficial effects on skeletal muscle and bone density [80].

Other positive effects of ghrelin are reported in patients with cachexia, sarcopenia (muscle wasting due to aging [342]), myopenia (muscle wasting due to chronic illness [343]), and frailty states [343,344]. Among the first applications of ghrelin in human chronic illness were studies in CHF [34] and COPD [78,345]. A pilot study in 10 CHF patients reported decreased levels of norepinephrine, improved cardiac function and exercise capacity [34]. In animal models using a placebo control the cardiac effects could not be confirmed [346], but these models validated weight gain and the skeletal muscle anabolic effects of ghrelin and ghrelin analogs [110,347,348]. Of interest, in CHF, ghrelin secretion is modulated by application of brain natriuretic peptide, which is produced in the heart and generally increased in heart failure [349]. In advanced CHF, ghrelin resistance has been observed [350]. The first studies of ghrelin in COPD, focused on cachexia [78]. Preliminary studies suggested that ghrelin increases skeletal muscle mass and improves exercise capacity. In a recent double-blind controlled trial, ghrelin improved symptom scores and increased respiratory muscle strength [345]. An additional indication for ghrelin treatment may be replacement therapy to patients who have undergone total gastrectomy due to gastric cancer. Theoretically, this is based on the fact that most ghrelin is produced in the stomach and that total gastrectomy results in loss of appetite, body fat and also lean body mass. Indeed, proof of concept studies in mice and humans have yielded positive results [224,225].

Inhibition of GOAT-mediated ghrelin acylation is considered an interesting opportunity to tackle obesity. Several GOAT inhibitors have been developed [60,62], and their intraperitoneal administration promotes weight loss while improving glucose tolerance in wildtype (wt) mice but not in mice deficient for ghrelin [60]. Ghrelin receptor agonists such as BIM-28131 (a.k.a. RM-131) have sustained effects in increasing body weight at long-term [351]. Chronic therapy with ghrelin agonists, however, is associated with weight gain, fat attrition, and insulin resistance. Such observations have led to drug discovery efforts designed to block ghrelin action, inducing a negative energy balance with a goal of treating obesity and insulin resistance. Although ghrelin levels are lower in obesity, that circulating ghrelin levels increase during a negative energy state may suggest that a method inhibiting ghrelin activity may be useful for preventing weight regain after diet and exercise (or another weight loss treatment) rather than as a weight loss therapeutic. The greatest utility of des-acyl ghrelin, in fact, appears to be for the treatment of insulin resistance, but only when injected in combination with ghrelin.

Several synthetic ghrelin mimetics are being pursued in clinical trials for diverse indications (Table 1). Three compounds are currently in development. Macimorelin is in clinical trials for the diagnosis of GH deficiency, relying on the stimulation of the hypothalamic pituitary axis, as described earlier in this review [352]. A second compound, Anamorelin, is in clinical trials for the treatment of cancer cachexia in the treatment of non-small cell lung cancer. Anamorelin mechanistically relies on the anabolic effects noted with ghrelin and ghrelin mimetics [353]. Ghrelin and synthetic ghrelin mimetics can also stimulate gastric emptying and can function as gastrointestinal prokinetics [354–357]. While our understanding of the mechanism by which ghrelin elicits these effects is still limited, a direct effect of the ghrelin agonist Relamorelin has been shown on human and mouse fundus and jejunum smooth muscle cells and human and rodent colonic circular smooth muscle [357], where expression of the GHSR has been described [358]. The ghrelin mimetic Relamorelin (also known as RM-131) is being developed for the treatment of diabetic gastroparesis [357], for which the compound is well tolerated, and has direct beneficial effects on gastric emptying, while reducing the incidence of nausea and vomiting. Beneficial effects have also been noted for colonic motility disorders where prokinetic effects have been demonstrated in humans in the colon [354]. Relamorelin is currently in Phase II clinical trials for treatment of diabetic gastroparesis and other gastrointestinal (GI) disorders [93]. In a rodent model of irritable bowel syndrome, ghrelin [359] and synthetic ghrelin mimetics [93] show improvements in tissue damage while modulating inflammatory responses. Synthetic ghrelin mimetics, therefore, may find beneficial applications in diverse functional gastrointestinal disorders.

## 8 Conclusions

Since ghrelin was discovered in 1999, we have come a long way in understanding ghrelin's multifaceted nature. Numerous studies on ghrelin's physiological effects (Figure 3) have led us to new aspects of human and animal physiology and revealed a complex system for acylating hormones, which was previously unknown. The next era should exploit this unique biology for diagnostic and therapeutic benefit.

## Figures and Tables

**Table 1:** Summary of ghrelin mimetics tested in clinical trials.

Compound	Company	Active/inactive	Indication
**Ghrelin mimetic**			
Pralmorelin	Kaken Pharma Sella Pharma	Approved Approved	Diagnostic for GH deficiency
Macimorelin	Aeterna Zentaris	Phase III	Diagnostic for GH deficiency
Anamorelin	Helsinn	Phase III	Anorexia/Cancer Cachexia
Relamorelin	Rhythm	Phase IIb	Diabetic gastroparesis
Ulimorelin	Tranzyme	Inactive	Opioid induced constipation/GI functions
Ipamorelin	Helsinn	Inactive	GI functional disorders
Carpromorelin	Pfizer	Inactive	Frailty in elderly
CP 464709	Pfizer	Inactive	Frailty in elderly
Tabimorelin	Novo Nordisk	Inactive	GH deficiency
Ibutamoren	Merck	Inactive	Frailty in elderly
Examorelin/Hexarelin	Diverse Academic sponsored studies	Inactive	GH release
SM 130686	Sumitomo	Inactive	Growth hormone deficiency
LY 426410 LY 444711	Eli Lilly	Inactive	GH release
